# Review of the Midbrain Ascending Arousal Network Nuclei and Implications for Myalgic Encephalomyelitis/Chronic Fatigue Syndrome (ME/CFS), Gulf War Illness (GWI) and Postexertional Malaise (PEM)

**DOI:** 10.3390/brainsci12020132

**Published:** 2022-01-19

**Authors:** James N. Baraniuk

**Affiliations:** Department of Medicine, Georgetown University, Washington, DC 20057, USA; baraniuj@georgetown.edu

**Keywords:** postexertional malaise, arousal, fatigue, threat assessment, anxiety, posttraumatic stress disorder, inferior colliculus, periaqueductal gray, midbrain reticular formation, parabrachial complex, locus coeruleus

## Abstract

Myalgic Encephalomyelitis/Chronic Fatigue Syndrome (ME/CFS and Gulf War Illness (GWI) share features of post-exertional malaise (PEM), exertional exhaustion, or postexertional symptom exacerbation. In a two-day model of PEM, submaximal exercise induced significant changes in activation of the dorsal midbrain during a high cognitive load working memory task (Washington 2020) (Baraniuk this issue). Controls had no net change. However, ME/CFS had increased activity after exercise, while GWI had significantly reduced activity indicating differential responses to exercise and pathological mechanisms. These data plus findings of the midbrain and brainstem atrophy in GWI inspired a review of the anatomy and physiology of the dorsal midbrain and isthmus nuclei in order to infer dysfunctional mechanisms that may contribute to disease pathogenesis and postexertional malaise. The nuclei of the ascending arousal network were addressed. Midbrain and isthmus nuclei participate in threat assessment, awareness, attention, mood, cognition, pain, tenderness, sleep, thermoregulation, light and sound sensitivity, orthostatic symptoms, and autonomic dysfunction and are likely to contribute to the symptoms of postexertional malaise in ME/CFS and GWI.

## 1. Introduction

Myalgic Encephalomyelitis/Chronic Fatigue Syndrome (ME/CFS) [1,2] and Gulf War Illness (GWI) [3,4] share features of post-exertional malaise (exertional exhaustion), fatigue that is not relieved by rest, unrefreshing and non-restorative sleep, total body pain, and systemic hyperalgesia and decreased quality of life. ME/CFS has been considered to be a chronic consequence following flu-like epidemics [5] but in general, has a sporadic heterogeneous presentation and unknown etiology. Prevalence is about 0.2 to 2% [6,7] and peak ages of onset are adolescence and 30s and 40s. There is a 3:1 to 4:1 female:male predominance. The 1994 Center for Disease Control criteria (“Fukuda”) require moderate to severe unremitting fatigue of new onset that persists longer than 6 months and has no explanation despite appropriate medical investigations plus at least four of the following eight ancillary criteria: cognitive complaints of short-term memory or concentration, sore throat, sore lymph nodes, myalgia, arthralgia, headaches including migraine, disordered sleep and post exertional malaise (PEM) (Table 1) [1]. Emphasis has been placed on PEM, the characteristically delayed exacerbation of the entire symptom complex following minimal physical, cognitive, or emotional efforts as a distinguishing feature of ME/CFS in the Canadian Consensus Criteria [2,8,9], Systemic Exertion Intolerance Disease (SEID) [10], 2020 Draft UK National Institute for Health and Care Excellence (NICE) guidance [11] and recent consensus statements in adults and pediatrics [12,13].

GWI continues to afflict the predominantly male cohort of 25 to 30% of veterans who were deployed to the 1990–1991 Persian Gulf War. The Centers for Disease Control criteria for Chronic Multisymptomatic Illness (CMI) require symptoms from at least two of three symptom clusters: general fatigue, mood and cognitive abnormalities, and myalgia/arthralgia (pain) [3]. Mood/cognitive symptoms can range from troubles with sleep, cognitive decrements, word fixing, anxiety, and depressive mood. An epidemiological comparison of symptoms between deployed and non-deployed Kansas veterans generated the Kansas criteria that require moderate or severe complaints in three of six domains: fatigue and sleep; pain; neurological/cognitive/mood; gastrointestinal; respiratory; and skin symptoms [4]. The etiology has been linked to exposures to neurotoxicants that were present in theatre including organophosphates, carbamates and other pesticides, sarin/cyclosarin nerve agents, and pyridostigmine bromide taken as a prophylactic medication against chemical warfare attacks [14,15]. The chronic symptoms are consistent with Chronic Organophosphate Induced Neuropsychiatric Disorder (COPIND) [16,17,18]. Psychiatric aetiologies have been ruled out [14].

Fibromyalgia has also had an evolution of criteria. The 1990 criteria required the symptom of widespread pain and sign of tenderness [19]. However, in 2010 the requirement for the physical sign of systemic hyperalgesia was removed and symptoms of fatigue, waking unrefreshed, cognitive difficulties, and somatic complaints were added [19,20,21]. As a result, many subjects with ME/CFS will qualify for fibromyalgia diagnosis. This becomes important in clinical trials for ME/CFS when fibromyalgia is listed as an exclusion but the exact definition of fibromyalgia is not provided. This aversion of pain is enshrined in the SEID criteria that focus on disability, fatigue, postexertional malaise, unrefreshing sleep, and cognition. Conversely, many of those defined by the 2010 fibromyalgia criteria may meet ME/CFS definitions. An alternative approach is to consider fibromyalgia to be a nociceptive disease with systemic hyperalgesia and allodynia, or to consider systemic hyperalgesia as another facet of ME/CFS that can be tested in clinical trials using multivariate regression analysis. Another implication is to consider the many diverse general symptoms of ME/CFS and GWI as interoceptive complaints emanating from visceral organs and tissues as a consequence of neuroplastic changes in sensory thresholds that lead to increased perception of discomfort and impairment of functioning. Postexertional symptom exacerbation may involve dynamic changes in these sensory thresholds with detrimental consequences called “fatigue” that disrupt normal brain activities such as restfulness, attention, working memory, and cognition.

A problem with subjectively defined criteria is the need to quantify symptom severity to ensure the correct diagnosis. This is especially apparent with older criteria that rely solely on fatigue as a symptom. They allow the inclusion of chronic idiopathic fatigue and atypical depression in clinical trials and, in contrast to modern criteria, do not explicitly require PEM, interoceptive, nociceptive, autonomic, and functional findings [22]. The imperfect vocabulary describing fatigue and exhaustion is another limit of subjective evaluations.

We approached this problem by developing a two-day submaximal exercise provocation paradigm to identify objective findings and infer pathophysiological mechanisms. The activation of brain regions at rest and during cognitive tasks was measured by functional magnetic resonance imaging (fMRI).

In general, when a task is performed, specific brain regions are required and they show task-specific patterns of correlated activation. When the task is finished, the task regions become deactivated and the brain is placed “at rest” with a different set of regions becoming activated “by default”: The default regions are in the medial prefrontal cortex, posterior parietal cortex, and precuneus, and they define the default mode network (DMN). Disruptions of the correlated network of activated brain regions at rest or during tasks are an indication of brain dysfunction.

The effect of exercise on the resting state was examined in ME/CFS and control subjects. Prior to exercise, ME/CFS had generally lower blood oxygenation level dependent (BOLD) signals than controls. However, after exercise, ME/CFS had elevated activation of the anterior node of the DMN in the medial prefrontal cortex compared to pre-exercise and to controls [23].

Next, brain activation was tested during a low cognitive load 0-back stimulus matching task (“see a letter, press a button”) in GWI. There was no difference between GWI and control subjects before exercise. However, following exercise, GWI had increased activation of the anterior DMN during the simple task [24]. Therefore, exercise caused dissociation of the DMN with aberrant anterior node activation in both ME/CFS and GWI. These dynamic changes in the medial prefrontal cortex may be objective evidence of postexertional malaise.

Then subjects were scanned during the difficult, high cognitive load continuous 2-back working memory task. Control, ME/CFS, and GWI were equivalent prior to exercise (baseline). However, postexercise ME/CFS had a significant increase in BOLD while GWI had a significant decrease in the dorsal midbrain, right middle insula, and left Rolandic operculum [25]. The region of interest in the midbrain extended from the left to right periaqueductal gray (PAG) and to the adjacent right midbrain reticular formation (MRF), inferior colliculus (IC), and lateral lemniscus, and caudally to the right lateral isthmus. The opposing directions of responses in ME/CFS versus GWI indicated different molecular mechanisms were perturbed by exercise in the two diseases.

**Figure 1:**
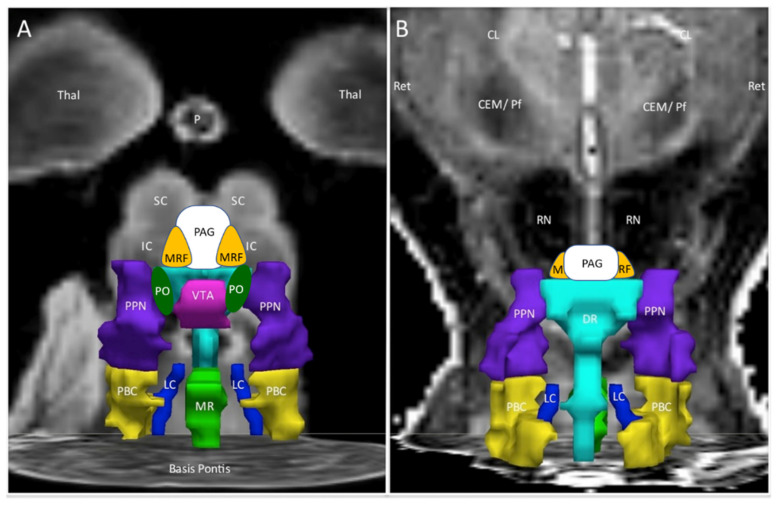
Ascending arousal network. The original figure [37] in the coronal plane was annotated by adding periaqueductal grey (PAG, white), midbrain reticular formation (MRF, orange), and pontis oralis (PO, dark green). (**A**) The anterior view showed the ventral midbrain with posterior thalamus (Thal) as a “ceiling”, the superior (SC) and inferior colliculi (IC) as the backdrop, and Basis Pontis as the “floor”. (**B**) The posterior view of the dorsal midbrain was oriented to show the plane of the centromedian/parafascicular nucleus (CEM/Pf), reticular nucleus (Ret), and central lateral nucleus (CL) of the thalamus, pineal (P), and midbrain red nuclei (RN). This depiction suggested three layers with the MRF and PAG being most rostral. The middle layer contained the ventral tegmental area (VTA, violet), bilateral pontis oralis (PO, dark green), and pedunculotegmental nuclei (L and R PTN, formerly pedunculopontine nuclei and labeled PPN in the original image, navy blue) and dorsal raphe (DR, cyan) in the posterior midline. The caudal layer had median raphe (MR, green) and DR flanked by bilateral locus coeruleus (LC, navy blue) and parabrachial complex (PBC, yellow). The nuclei are contorted around white matter tracts that were not part of the original region of interest but constitute much of the ventral midbrain. The orientations of the nuclei displayed here do not align perfectly with the Paxinos histological atlas of the human brainstem [38].

A seed region approach was then applied to investigate the potential extent of the exercise-induced changes and differences between groups. The Harvard Ascending Arousal Network atlas [26,27] was referenced as it was defined from histological sections and diffusion studies of brainstem white matter tracts (Figure 1) [26]. We proposed that affected nuclei may participate in the pathology of postexertional malaise based on the profound influences of these nuclei on threat assessment, pain, negative emotion, attention, wakefulness, and instinctual neurobehaviours. Evaluation of midbrain and brainstem was supported by exercise-induced autonomic dysfunction [28,29,30,31] and worsening of dizziness and lightheadedness in ME/CFS [29] as well as magnetic resonance imaging findings of brainstem atrophy in GWI [32,33] and dysfunction in ME/CFS [34,35,36].

The ventral half of the midbrain is dominated by white matter tracts. The superior cerebellar peduncles decussate in the medial midbrain and connect deep nuclei of the cerebellum to the midbrain, thalamus (cerebellothalamic tract), and red nucleus (cerebellorubral tract). Lateral to the cerebellar peduncles are the medial lemniscus that conveys sensory information from the spinal cord dorsal columns to the thalamus (spinothalamic tract) and the lateral lemniscus that carries auditory information from cochlear nuclei to the inferior colliculus. The anterior rim of the midbrain contains the cerebral peduncles with ascending nerve tracts to the thalamus and cortex, and descending corticospinal, corticopontine, and corticobulbar tracts. These major midbrain white matter tracts as well as smaller ones are relevant because of atrophy and dysfunction in ME/CFS [34,35,36,39] and GWI [32,33,40,41].

**Figure 2:**
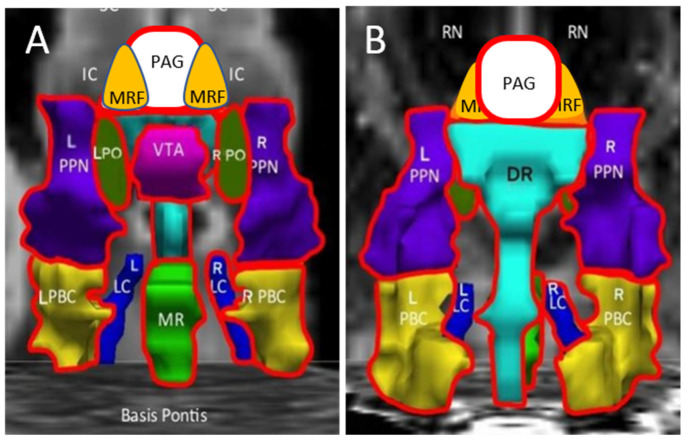
Incremental changes (∆) in the ascending arousal network nuclei. The red lines highlighted nuclei with significant exercise-induced incremental changes between postexercise and preexercise scans. ΔBOLD was significantly larger (more positive, increased activation) for ME/CFS compared to GWI (negative change, diminished BOLD) in the midline PAG, VTA, DR and MR, bilateral PBC, PO and PTN, and R_LC. Controls had no net changes. (**A**) is the anterior ventral view and (**B**) is the dorsal posterior view.

The differential effects of exercise were summarized by the incremental changes between pre- and postexercise days (Figure 2). ΔBOLD was significantly larger (more positive, increased activation) for ME/CFS compared to GWI (negative change, diminished BOLD) in the midline PAG (periaqueductal gray), VTA (ventral tegmental area), DR (dorsal raphe), and MR (median raphe), bilateral PBC (parabrachial complex), PO (pontis oralis) and PTN (pedunculotegmenal nuclei, formerly pedunculopontine nuclei, PPN), and R_LC (right locus coeruleus). Controls had no net changes with exercise.

## 2. Midbrain Nuclei

Significant differences in activation of the midbrain nuclei in the ascending arousal network (Figure 1 and Figure 2) have special implications for pain, negative emotion, and neurobehavioural dysfunction in ME/CFS and GWI [42]. Great advances have been made in learning the functions of this vital yet underappreciated organ. A review of each nuclear region provides a fresh perspective for interpreting the current results and generating new hypotheses for postexertional malaise, ME/CFS, and GWI.

## 3. Development of the Midbrain and Isthmus

**Figure 3:**
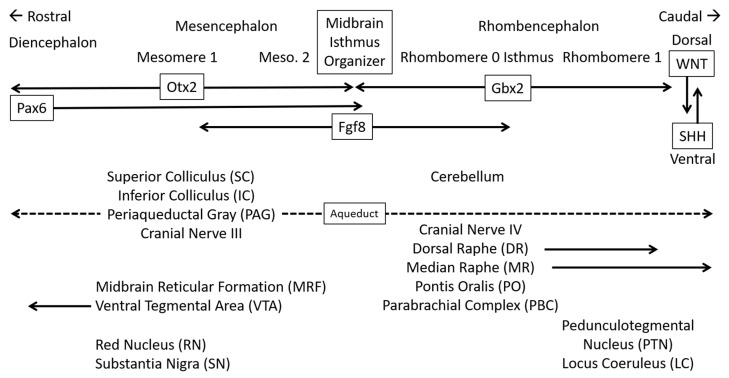
Embryology and general structure of midbrain and proximal brainstem. The cartoon is oriented with rostral on the left (towards the nose), caudal on the right (towards the tail), dorsal (posterior) at the top, and ventral (anterior) to the bottom. Specific patterns of growth factors secreted during development control the differentiation of every brain region. Otx2 controls the development of the diencephalon and mesencephalon. Gbx2 regulates the rhombencephalon. Pax6 from the diencephalon diffuses to control mesencephalon development but does not affect the rhombencephalon. The midbrain isthmus organizer produces Fgf8 that diffuses to regulate rostral and caudal development. The organizer does not have an anatomical remnant but forms the boundary between mesencephalon and rhombencephalon. Gradients of Wnt and Shh control dorsal-ventral development. The orthogonal diffusion patterns produce a three dimensional checker board of growth factors at various concentrations around the central aqueduct (dashed line) that control gene expression profiles to induce the localized development of each specific nucleus. Mesomere 1 is larger than mesomere 2 and is the source of the midbrain nuclei including cranial nerve III. The ventral tegmental area (VTA) extends into the diencephalon (arrow). The isthmus (rhombomere 0) is the origin of the cerebellum and cranial nerve IV. The midbrain reticular formation (MRF) and pontis oralis (PO) are part of the reticular activating system that extends to autonomic and motor centers in the medulla.

The anatomical organization of the brainstem has been invigorated by developmental studies of organogenesis. Midbrain and isthmus (proximal or rostral brainstem) regions are now defined from prosomeric genoarchitectonics that are based on embryonic vesicles of the neural tube [43,44,45]. Specific patterns of growth factor secretion control the development of every brain region. The division between the embryonic mesencephalon and rhomboencephalon is defined by rostral Otx2 (Orthodenticle Homeobox 2) and caudal Gbx2 (Gastrulation Brain Homeobox 2) gene expression (Figure 3) [46]. The boundary is called the midbrain-isthmic organizer. Otx2 directs the formation of the rostral midbrain and oculomotor nuclei in mesomere 1 and the thin sliver of the more caudal mesomere 2. Gbx2 induces the formation of the hindbrain beginning with the isthmus in rhombomere 0. Cells in the midbrain-isthmic organizer secrete Fgf8 (Fibroblast Growth Factor 8) that diffuses in rostral and caudal directions to form gradients that control rostral-caudal differentiation and development of nuclei [47]. Pax6 (Paired Box 6) is secreted from the diencephalon and controls midbrain development by antagonizing signals from the isthmic organizer.

The dorsal-ventral organization is determined by gradients of SHH (Sonic Hedgehog Signaling Molecule) from the ventral floor to basal plate, and WNT (Wingless-Type MMTV Integration Site) family proteins from the dorsal roof and alar plates. WNT1 is most influential in the dorsal midbrain.

The gradients of growth factors generate dorsal-ventral and rostral-caudal patterning axes that govern the differentiation of specific nuclei and influence instinctual defensive and other behaviors such as aggression versus passivity [48]. The complex interactions may provide epigenetic imprints on dorsal midbrain gene expression in a way that makes this region particularly susceptible to as yet uncharacterized toxic molecular injuries that are responsible for distinct molecular deficits or neurobehavioural patterns in GWI and ME/CFS.

Midbrain mesomere 1 is the origin of the inferior colliculi, bilateral midbrain reticular formation, PAG, and oculomotor cranial nerve [43,45]. The isthmus (rhombomere 0) is the origin of a trochlear motor nucleus (cranial nerve IV), parabrachial complex (PBC), and pontis oralis (PO). The dopaminergic ventral tegmental area (VTA) extends from the isthmus to the diencephalon. Rhombomeres 0 and 1 form the midline serotonergic dorsal raphe (DR), caudal linear, and central or median raphe (MR) nuclei. Rhombomere 1 is the origin of the cholinergic pedunculotegmental (PTN, formerly pedunculopontine nucleus, PPN) and noradrenergic locus coeruleus (LC).

These small regions are a challenge to differentiate by MRI methods [49,50]. It is difficult to map the embryological and anatomical nuclei to (x,y,z) MRI coordinates such as Montreal Neurological Institute (MNI) because of the extensive flexion of the neural tube that generates the wedge-shaped midbrain with wide dorsal and narrow ventral surfaces, and angulation of the brainstem at the base of the skull. The challenge is increased given the degree of motion of the brainstem with each heartbeat [30,31,51,52]. However, the results generate the hypothesis that different nuclei are differentially activated during the working memory tasks and are altered by exercise [25]. The outcomes can be used for sample size calculations for future studies.

The cerebellum vermis and hemispheres develop from the dorsal isthmus (isthmocerebellar hindbrain). Subsets of GWI veterans who were defined based on postural tachycardia and changes induced by exercise had different patterns of activation in the vermis and cerebellar hemispheres after exercise [53]. This piqued our investigation of midbrain nuclei and dynamic changes with postural tachycardia. The normal response to standing up is an increase of 10 to 15 beats per minute that is not affected by exercise; this defined our STOPP (Stress Test Originated Phantom Perception) or “normal” subgroup [54]. At the other extreme is postural orthostatic tachycardia syndrome (POTS) that had increases of ≥30 beats per minute when standing that did not change with exercise [29,55]. The newly discovered START subgroup (Stress Test Activated Reversible Tachycardia) [53,54] had normal changes in heart rate before exercise but developed significant postural tachycardia after exercise. The effect was transient as it lasted 1 to 3 days. The START phenotype showed a unique decrease in BOLD activation in R_LC and bilateral PBC following exercise.

## 4. Periaqueductal Gray

The periaqueductal gray (PAG) is a profuse plexus of small and medium-sized neurons and largely unmyelinated fibers at the heart of the midbrain surrounding the central aqueduct. PAG is the hub for instinctive behavioral and social reactions that drive emotional experience and physiology in response to threats. It provides motivations for hunting, foraging, sexual and maternal actions. Subdivisions have distinct connectivity for pain modulation, interoception (visceral sensations such as dyspnea and gastrointestinal discomfort), and executive decision-making functions [56,57]. The dorsal columns convey efferent axons from the more rostral “medial defense zone” made up of premammillary nuclei, amygdala, stria terminalis, hippocampus, and lateral septum [58]. This system has been implicated in survival, threat analysis, freeze and flight responses, and in models of anxiety, panic disorder, and rumination [59,60]. Exteroceptive perception identifies novelty in the environment that may be a risk or opportunity (e.g., predator or prey). PAG participates in threat assessment by making instantaneous decisions across the spectrum of existential risks ranging from predator vs. prey status, the proximity of a threat, social hierarchy interactions, foraging vs. feeding prospects and generates visceromotor responses to deal with the perceived threat [61]. Of particular relevance to ME/CFS and GWI, the PAG processes and integrates pain, negative emotion such as sadness and anger [62], autonomic control [49,63,64], vigilance, arousal, attention [65], memory, and language that are essential for cognitive and social purposes.

Perception of a novel stimulus as a social inferior or feeding opportunity leads to “instinctual” predator-like behavioral responses with displays of social superiority or attacks on edible prey [48]. The perception that the novelty is a predator leads to prey-like behaviors that include danger detection (i.e., risk assessment) and contemplation of escape strategies. Threat detection activates the PAG and midbrain reticular formation to transition from relaxed wakefulness to high general attention [66] that allows for focused interrogation of threats (e.g., freezing in place) to gauge if the danger is remote or imminent [61]. Arousal leads to sympathetic activation for imminent action. Freezing in place with conscious tonic immobility may be the appropriate action if the movement would betray one’s position or lead to detection by a nearby predator. Freezing with a defensive approach may allow time to collect more information when assessing an ambiguous threat; deliberation without decision making and further action has been considered to be a model of rumination [59,60]. If the novelty is perceived as a threat, then flight is triggered via an escape route or to shelter. A defensive attack is infrequent, but may be precipitated if the predator is within a dangerous distance and escape is not possible (“backed into a corner”) [67,68]. When a threat becomes inescapable (i.e., “in the jaws of death”) [61], the ventrolateral PAG may activate the parasympathetic nervous system and override sympathetic influences leading to a state of hypoarousal referred to as defensive freezing behavior, submissive shutdown response, or conservation withdrawal [69]. A defensive freezing circuit has been defined by optogenetic and neuroanatomical tracing methods in animal models. The inhibitory pathway originates in the central nucleus of the amygdala and extends to the ventrolateral PAG where disinhibition of excitatory outputs to pre-motor targets in the magnocellular nucleus of the medulla leads to the statuesque freezing of muscular posture [69]. Dysregulation of this survival circuit in humans has been implicated in anxiety-related disorders. Other passive defensive actions include social submissiveness and faint with death simulation (collapse immobility or unconscious flaccid paralysis) [70]. The defensive behaviors may have similarities to sickness behaviors and quiescent immobility that promote rest and healing. Misinterpretation or dysregulation of these instinctual decision-making processes may contribute to anxiety, chronic pain [71], autonomic disorders [72], and posttraumatic stress disorder (PTSD) [72].

An alternative response to a threat is found in the “Tend and Befriend” hypothesis in females [73] with prosocial activation in stressful social settings [74]. The prefrontal cortex, oxytocin, female reproductive hormones, and endogenous opioid peptide mechanisms may contribute to these nurturant activities that protect the self and offspring, promote safety, reduce distress, and aid in the creation and maintenance of social networks. Tend and befriend and freeze fight-flight faint may represent graded responses to stressors that are dysfunctional in vulnerable individuals at risk for negative affect, PTSD, ME/CFS, or GWI.

Posttraumatic stress disorder (PTSD) is a state of event-induced affective and recall dysfunction with hyperarousal and active fight-flight defensive responses. PTSD was found in 45% of the current GWI sample. Memories of the specific inciting event lead to stereotyped symptoms. A dissociative subtype is characterized by passive or submissive defensive responses, depersonalization, and autonomic blunting. Functional connectivity studies in control subjects showed a link for the dorsolateral PAG to the left cerebellar lobule IV, but none for the ventrolateral PAG [75]. Nondissociative PTSD patients demonstrated greater connectivity of dorsolateral and ventrolateral PAG with the dorsal anterior cingulate cortex (dACC), right anterior insula, orbitomedial prefrontal cortex, and bilateral fusiform gyrus that are involved in salience, visual recognition of fearful faces, hyperarousal, sympathetic activity, cessation of current tasks with switching to initiate active coping strategies such as fight and flight. The dorsolateral PAG had functional connectivity with cerebellum lobule VI that processes fearful faces and is involved in the startle response [76]. Sympathetic effects are mediated by efferent autonomic pathways from the medulla to spinal neurons that innervate peripheral ganglia connected to vascular, visceral, and adrenal medulla target organs. Dissociative subjects had functional connectivity of both dorsolateral and ventrolateral PAG with orbitomedial prefrontal cortex, left fusiform gyrus, and cerebellum lobule VI, as well as predominantly dorsolateral connectivity to the left precentral and postcentral gyri. In comparing the two subtypes, dissociative subjects had greater connectivity between the ventrolateral PAG and right temporoparietal junction, right Rolandic operculum, left fusiform gyrus, and cerebellum lobule VI. The right temporoparietal junction and Rolandic operculum are associated with discrimination of self vs. non-self and body consciousness that may be relevant to emotional detachment from traumatic memories during states of depersonalization.

Connectivity of these regions in PTSD is reminiscent of our previous findings in the midbrain, right middle insula, and left Rolandic operculum during the postexercise cognitive task where ME/CFS had significantly elevated BOLD but GWI had decreased signal [25]. When all data were reviewed, ME/CFS had a trend for lower BOLD than control and GWI preexercise, while GWI was lower postexercise. The postprovocation data suggest the hypothesis that global cerebral blood flow was altered by exercise but in opposite directions in ME/CFS (elevated towards control levels) in contrast to GWI (reduced after exertion). These data were consistent with reduced cerebral blood flow in ME/CFS [77,78,79] and alterations in neurovascular coupling in GWI [80,81].

PAG regulates respiration by receiving and interrogating processed sensory and motor information from limbic, prefrontal, and anterior cingulate cortex regions and relaying the results to respiratory control centers in the caudal brainstem [82]. The ventromedial medullary tegmentum and nucleus retroambiguus provide motor systems that control abdominal and intrathoracic pressures used to generate vocalization, coughing, sneezing, and vomiting [83]. Dysfunction of these pathways may contribute to dyspnea [84], upper airway difficulties that are common complaints in ME/CFS and GWI [85], perceptions of choking as in globus hystericus, and explosive diarrhea in GWI. Projections to the pelvic organ stimulating center and the pelvic floor stimulating center coordinate actions of the pelvic organs, bladder, uterus, prostate, seminal vesicles, distal colon, and rectum in parturition, urination, ejaculation, and defecation. Dysfunctional regulation may contribute to irritable bowel and bladder complaints.

The PAG and midbrain reticular formation (MRF) regulate pain perception and tenderness. The PAG stimulates descending anti-nociceptive pathways to the rostral ventromedial medulla (RVM) and spinal cord dorsal horn that induce endogenous opioid-based analgesia. The analgesic effect is stronger in men than women indicating a sexual dimorphism [86,87]. Conversely, midbrain dysfunction increases pain perception by contributing to windup, allodynia, and systemic hyperalgesia [88]. Sexual dimorphism of midbrain nociceptive and descending PAG anti-nociceptive circuits is consistent with the exquisite tenderness of GWI females and intermediate sensitivity of ME/CFS women [89] compared to controls when tested by pressing a dolorimeter against their skin [90]. Deep brain stimulation of the PAG has been suggested to release endogenous opioids for the relief of pain [91] and may have therapeutic potential in ME/CFS and GWI.

## 5. Inferior Colliculus (IC)

The inferior colliculi are in the dorsal tectum of the midbrain and had significant changes in fMRI activation after exercise in ME/CFS and GWI [25]. The IC are innervated by the ascending hindbrain auditory pathway (the lateral lemniscus) and somatosensory pathways from the pons and medulla as well as PAG, VTA, substantia nigra, LC, DR, and PTN [92]. This information is integrated and relayed to the superior colliculus (visual integration), pretectum, PAG, and medial geniculate body of the thalamus. The nucleus participates in multimodal sensory perceptions, vestibulo-ocular reflex, predator aversion and escape, prey localization, social communication, analgesia, and fear-related behaviors. Activation of the inferior colliculus enhances perception and alertness by decreasing attentional thresholds that promote general “attentional focusing” in thalamocortical and pontocerebellar circuits [93]. Acoustic stimulation acutely increases attention and surveillance leading to a visual and truncal orientation towards the sound and vestibular stimuli with active eye movement mediated by cranial nerve III (oculomotor). Other effects include generalized hyperarousal and aversive behaviors such as the startle response [94,95,96,97]. Light and sound sensitivity and the startle response are accentuated in ME/CFS, GWI, and veterans with PTSD [97]. Inferior colliculus contributes to the V wave of brainstem auditory evoked potentials (BAEP) [98]; we predict exertion will alter brainstem potentials in ME/CFS and GWI and be an indicator of PEM.

The inferior colliculus is highly metabolically active with exceptional demands for blood flow and oxygen for aerobic mitochondrial metabolism making it vulnerable to toxic and traumatic brain injury [92]. The organ has a particular requirement for phosphorylated thiamine (Vitamin B1) in the synthesis of glutamate and GABA neurotransmitters and as a cofactor in the tricarboxylic acid (TCA, Krebs, or citric acid) cycle. These metabolic vulnerabilities make the inferior colliculus susceptible to dysfunctional pathology in alcoholism, Wernicke-Korsakoff Syndrome, heavy metal poisoning, and Leigh’s syndrome. For example, ethanol is incorporated into the TCA cycle as acetate and derails glycolysis as an energy source. This provides a clue to alcohol sensitivity in ME/CFS. Given the deficient pyruvate metabolism in ME/CFS [99], it is possible that ethanol may be inadequately metabolized to acetate and may lead to ethanol or acetaldehyde neurotoxicity that contributes to alcohol aversion in ME/CFS. The subtle early symptoms of Wernicke–Korsakoff Syndrome include fatigue, headache, concentration difficulties, irritability, and abdominal discomfort [100] and have superficial similarities to GWI and ME/CFS. This suggests the hypothesis that high demands for aerobic metabolism and thiamine in the inferior colliculus contribute to GWI and ME/CFS pathologies.

Cerebral blood flow is decreased in ME/CFS during heads up tilt [78] and exercise [101] which may reduce oxygen supply to the inferior colliculus leading to dorsal midbrain dysfunction. Exercise-induced lability of cerebral blood flow and neurovascular coupling may contribute to the dysfunctional BOLD patterns in the midbrain, insula, and cerebellum vermis in GWI and ME/CFS [25] but with different mechanisms and statistically reciprocal outcomes in the two diseases.

## 6. Oculomotor Nerve Nuclei

The oculomotor nucleus is ventral (anterior) to the PAG in the midbrain. This complex includes the main motor nucleus that innervates extra-ocular muscles, decussation of the crossed component of the oculomotor nerve that directs eye muscles to gaze upwards, and the parasympathetic preganglionic accessory (Edinger-Westphal) nucleus that innervates the ciliary ganglion and controls intra-ocular involuntary smooth muscles of the pupil (miosis) and ciliary body (accommodation). Exercise may have induced dysfunction of the midbrain oculomotor and isthmus trochlear nerves that were combined with dyscoordination in the inferior colliculus (auditory)–superior (visual) colliculus orientation system leading to visual dysfunction in ME/CFS [102,103]. Visual disruption is predicted to be a component of postexertional malaise based on the proximity of these nerves to the midbrain region of interest, significant changes in BOLD following exercise, and dysfunctional accommodation and visual changes in ME/CFS and GWI.

The trochlear or fourth cranial nerve arises in the isthmus.

## 7. Midbrain Reticular Formation (MRF)

Immediately anterior and lateral to the PAG are the bilateral midbrain reticular formation (MRF). The MRF is the rostral terminus of the reticular activating system that extends caudally into the isthmus as the “reticularis” pontis oralis (PO) and through the pons to the medulla. The MRF are traversed by spinothalamic, trigeminothalamic, and medial lemniscus pathways and may survey and modulate their information flow. These filtering and integration functions during arousal are in contrast to the pontine and medullary reticular formation that receives sensory input from cranial nerves and the spinal cord and have premotor efferent autonomic control of visceral activities. The mesencephalic reticular formation controls upwards gaze through projections to the nearby Edinger–Westphal nuclei. Horizontal gaze is controlled by the pontine region, while the medulla regulates head movements and gaze holding. In humans, the MRF is activated during the transition from a relaxed awake state to an attention-demanding state during reaction-time tasks [66] and focused interrogation of threats while interpreting the proximity of danger (e.g., freezing in place to assess danger) [61]. Midbrain lesions are associated with hypersomnia. Activation of the cuneiform region that is near the PO are correlated with cardiovascular dysfunction in ME/CFS [34,104].

**Figure 4:**
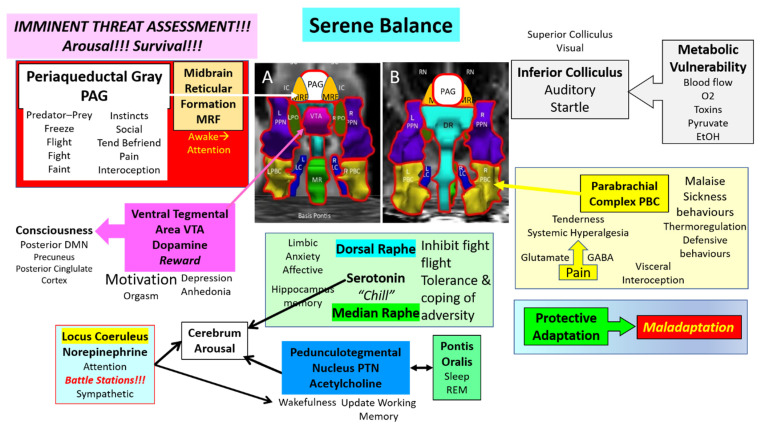
Midbrain ascending arousal network nuclei. The primary goal of the midbrain nuclei is to rapidly and accurately assess visual, acoustic, and other exterioceptive sensory inputs for threat analysis, to determine motivations in predator-prey and social settings and organize stereotyped “instinctual” responses for survival. The outputs to other neural centers help to maintain the balance for viable homeostasis. Chronic stressors can lead to protective adaptation that maintains homeostasis. Excessive stressors lead to maladaption of the nuclei and dysfunction of the arousal network.

Although the MRF, PAG, and other nuclei are known for their effects under dysfunctional conditions, their normal role is to maintain safe vigilance and protective responses to sudden unexpected stimuli (Figure 4). Other nuclei play important roles in shaping responses to the normal stressors of everyday life and occasional severe stress exposures. The system has the capacity for protective adaptation during stressful periods to maintain a healthy balance despite the taxing times.

## 8. Nuclei with Amines as Predominant Neurotransmitters

Aminergic nuclei in the ventral midline are the serotonergic median raphe (MR) and dorsal raphe (DR) and dopaminergic ventral tegmental area (VTA). Bilateral nuclei in the isthmus include the adrenergic locus coeruleus, co-localized cholinergic and nitric oxide synthase positive pedunculotegmental nuclei (PTN), pontis oralis (PO), and parabrachial complex (PBC). These nuclei modulate the flow of ascending sensory afferent information from cranial nerves and spinothalamic tracts as they project to more rostral structures. Efferents to the cerebrum release aminergic neurotransmitters in the cortex that increase arousal, attention, and focus. The reverse of these processes contributes to refreshing sleep. Unrefreshing sleep is a critical element of ME/CFS, GWI and FM. Cholinergic nuclei were of interest because GWI veterans were exposed to irreversible acetylcholinesterase inhibitors such as the nerve agent sarin, organophosphates, carbamates, and pesticides that may have caused long-lasting neurotoxic disruption of cholinergic pathways and functions [14,15].

## 9. Locus Coeruleus (LC), Norepinephrine and Sympathetic Activation

Locus coeruleus (LC) is the predominant source of noradrenergic innervation in the brain [105,106]. Descending afferent inputs to the LC are received from insula, central nucleus of the amygdala, dorsolateral and dorsomedial prefrontal cortex (PFC), hypothalamic paraventricular nucleus (PVN), and suprachiasmatic nucleus (SCN) to provide circadian-based regulation of arousal. Local midbrain connections are received from PAG and raphe nuclei, and bidirectional communication with the ventral tegmental area (VTA) that modulates reward and depressive phenotypes. Ascending tracts from rostral, ventrolateral, and dorsomedial medulla regions participate in the regulation of sympathetic control and behavioral orienting.

Efferent adrenergic tracts from the locus coeruleus ascend to the periventricular nucleus and supraoptic nucleus of the hypothalamus for autonomic and endocrine regulation; ventral and central nucleus of the amygdala for salience detection and associative learning; the “diagonal band”, medial septum, and hippocampus that influence learning, memory and plasticity; and corpus callosum to diffusely innervate the cingulum and cerebral cortex for regulation of attention, arousal and the cognitive evaluation of pain. These efferent projections from adrenergic as well as cholinergic nuclei widely innervate the midbrain, subcortical and cerebral cortex and promote wakefulness, arousal, general attention, and focused concentration [107]. Local projections innervate the PAG, tegmentum, raphe nuclei, and VTA. The LC promotes wakefulness by releasing norepinephrine from diffuse efferent varicosities and endings in the cerebral cortex. In general, the LC inhibits the PTN but may stimulate wakefulness-promoting cholinergic neurons of that nucleus.

The LC is the major premotor sympathetic autonomic nucleus. LC increases peripheral sympathetic activity by the release of norepinephrine for activation of α1-adrenoceptors on preganglionic sympathetic neurons in the spinal cord. LC projections reduce parasympathetic activity via α2-adrenoceptors on preganglionic parasympathetic neurons in the vagus dorsal motor nucleus, salivatory nuclei, and nucleus ambiguous. The LC acts as a tripwire for threat assessment and instinctual responses such as freeze-fight-flight-faint by integrating sensory, PAG, amygdala, and other inputs resulting in instantaneous responses for focused cerebral attention and sympathetic activation for immediate action. Inappropriate dysfunctional activation of this circuit contributes to anxiety and PTSD [72]. Atrophy of the right locus coeruleus was found at autopsy in veterans with PTSD [108].

Noradrenergic locus coeruleus neurons also express the neuropeptide galanin [109]. This 29/30 amino acid peptide neurotransmitter acts on three receptors GalR1, -R2, and -R3. Galanin is released with norepinephrine from neuron terminals during arousal and other activities and binds to inhibitory autoreceptors that provide a brake to prevent overexcitation by norepinephrine on the postsynaptic cells. Under ordinary circumstances, galanin may provide protective resilience to stressors when there is situational excessive LC activation. Chronic stressors may increase the expression of galanin as suggested by postmortem studies of depressed patients who committed suicide. They had upregulation of mRNA transcripts for galanin and GalR3 in the LC with a parallel decrease in DNA methylation of the genes that implicated epigenetic mechanisms in the pathology of affective disease. It is conceivable that a similar mechanism acts in PTSD. The result would be overexpression of galanin compared to norepinephrine with a relative decrease in arousal, cognitive effort, and other activation states. Galanin antagonists may have antidepressant activity. 

Sympathetic effects can be overridden by parasympathetic Type B myelinated vagal fibers emanating from the ventral nucleus ambiguous. Unmyelinated Type C fibers from the dorsal motor nucleus of the vagus induce a state of immobilization [110,111]. Parasympathetic signals are conveyed to the periphery via the Xth cranial nerve as opposed to the spinal cord that conveys sympathetic reflexes.

## 10. Dorsal Raphe (DR), Median Raphe (MR) and Serotonin (5HT)

The dorsal raphe (DR) [112] and more caudal median raphe (MR) are ventral to the PAG and extend into the hindbrain. These nuclei contain serotoninergic neurons that play important but differential roles in mood regulation through projections to limbic regions [113]. DR neurons project to limbic forebrain regions involved in stress, conflict, and anxiety-provoking processes and are relevant to anxiety and affective disorders. Serotonergic neurons in the lateral portions of the DR inhibit centers that initiate and promote fight-or-flight responses and may be relevant to panic disorder. MR tracts are involved in tolerance and coping with aversive stimuli, which may be relevant to affective disorders such as depression. MR neurons project extensively to the hippocampus and so likely play a role in the formation of long-term memory. The raphe nuclei promote wakefulness by activating the cerebral cortex via 5HT2A receptors.

## 11. Ventral Tegmental Area (VTA) and Dopamine

The dopaminergic ventral tegmental area (VTA) extends from the midbrain to diencephalon. It participates in the natural reward circuitry of the brain to promote task-oriented motivation, associative learning, positive emotions, and orgasm [114,115]. Dysfunction leads to psychiatric disorders and may contribute to anhedonia. The functions of dopamine are regulated by inputs from many limbic and arousal centers. Glutamatergic afferents from the prefrontal cortex, hypothalamus, superior colliculus, PAG, DR, PTN, and other areas increase the firing rates of the dopamine neurons in the VTA. Glutamatergic neurotransmission is essential for the effects of drugs of abuse. GABA (gamma-aminobutyric acid) inputs from the DR, PAG, nucleus accumbens, ventral pallidum, and other regions inhibit dopamine release. Cholinergic inputs from the PTN modulate reward circuits. The VTA promotes wakefulness via activation of the LC.

Human consciousness is reliant on the functional connectivity of VTA with the precuneus and posterior cingulate cortex nodes that form the posterior hub of the default mode network (DMN). The DMN mediates self-referential thought, “mind-wandering”, systems planning, and abstraction of negative emotion when there are no tasks to be performed [50,116]. Exercise modifies the activity of the anterior node of the DMN in the medial prefrontal cortex with increased activation during a low cognitive load test in GWI compared to control [24], and during rest in ME/CFS [23]. Disruption of connections between the VTA-precuneus-posterior cingulate cortex circuit leads to disorders of consciousness [117]. The “dopamine brainstem disconnection” hypothesis has important implications for cognition and sensations of “brain fog” and unrefreshing sleep in ME/CFS and GWI that may become amenable to future therapeutic interventions [118].

The midbrain dopaminergic (DA) system includes the VTA and substantia nigra pars compacta (SNc) which are not part of the arousal network [119]. Subsets of dopaminergic neurons with distinct patterns of gene expression and electrophysiologic properties respond rapidly to sensory cues relevant to action selection, motor performance, motivation, and reward-based learning, working memory, and cognition. Most neurons respond to reward stimuli by increasing their activity and are inhibited by aversive stimuli. This differential process provides coding for motivational value when learning reward-oriented behavior. Neurons that enact motivational value are located in the VTA and medial SNc and project primarily to the nucleus accumbens and ventromedial prefrontal cortex. They support Pavlovian learning of actions that lead to successful outcomes. Dorsolateral SNc neurons code for motivational salience and project to the caudate, dorsomedial striatum, and dorsolateral prefrontal cortex to modulate goal-oriented behavior. Neurons in the ventral tier of the SNc project to the dorsolateral striatum (putamen) and regulate habit formation and automatic motor behavior. The ventral SNc neurons are vulnerable to oxidative injury; neurodegeneration leads to Parkinson’s disease (PD). Nonmotor actions of VTA and SNc contribute to depression, cognitive dysfunction, and impaired impulse control. The SNc is outside the region of interest found in ME/CFS and GWI [25] but may participate in the Parkinsonian complications of GWI [120].

## 12. Pedunculotegmental Nuclei (PTN), Pontis Oralis (PO), and Acetylcholine

Bilateral pedunculotegmental (PTN) and pontis oralis (PO) are cholinergic nuclei in the isthmus. PTN modulate descending motor and emotional information from the basal ganglia and ascending hindbrain afferent and regulatory signals destined for the thalamus. They contribute to attention and wakefulness. The cholinergic neurons dynamically update the current behavioral state in response to ongoing environmental changes and simultaneously update new contextual associations and restrain obsolete past actions [121]. These activities were put to use in our continuous 2-back working memory task. Attention, arousal, and wakefulness may be regulated by reciprocal interactions between inhibitory PTN GABAergic effects on the PO that suppresses sleep signals and generate wakefulness [122]. Reduced PTN activity may indicate active sleep, while reduced PO activity may foster active wakefulness. Reduced activity of the locus coeruleus (LC) in PTSD may lead to reduced adrenergic inhibition of the PTN that permits an increase in anxiety, hyperarousal, and drive for rapid eye movement (REM) sleep with nightmares and traumatic memories (flashbacks). The PTN also participates in the regulation of posture and locomotion, gait instability, falling, and voluntary movement [123].

Although a part of the reticular activating system, the pontis oralis (PO) provides cholinergic signals to generate REM sleep. Disruption of the balance may lead to insomnia and unrefreshing sleep.

Stress in animal models induces corticosteroids and neurosteroids that activate the PO and other nuclei leading to tonic immobility [124].

## 13. Parabrachial Complex (PBC)

The parabrachial complex (PBC) has functions that are aligned with ME/CFS and GWI diagnostic criteria of malaise, pain, tenderness, and perception of fever. The complex is directly caudal to the PTN on the surface of the superior cerebellar peduncles in the dorsal isthmus. PBC is a general primary hub to receive, process, and relay somatic and visceral nociceptive signals from the dorsal horn of the spinal cord and cranial nerve sensory areas. The primary nociceptive input is directly from neurokinin 1 (substance P) receptor-expressing neurons in the superficial dorsal horn that are dysregulated in inflammation or nerve injury and contribute to persistent pain, hyperalgesia, and allodynia [125]. Pain and visceral sensations interrogated by PBC are forwarded to the PAG, thalamus, hypothalamus, and amygdala for further information processing [126].

Glutamatergic neurons in the lateral PBC relay noxious information to the amygdala for processing affective responses. Optogenetic activation of excitatory glutamatergic neurons induces neuropathic pain-like behavior in naïve mice while inhibition of glutamatergic neurons alleviates basal nociception and neuropathic pain induced by peripheral nerve injury [127]. Conversely, activation of inhibitory GABAergic neurons has no effect on basal nociception but alleviates and may prevent neuropathic pain-like behaviors. It is proposed that disruption of the delicate balance between excitatory glutaminergic and inhibitory GABAergic neurons in the PBC may underpin the chronic pain and systemic hyperalgesia in GWI and ME/CFS [89].

PBC is recruited in states of malaise as an adaptive component of the sickness response [128], and so may participate in the experience of postexertional malaise in ME/CFS. Perception and control of body temperature are mediated by PBC subnuclei in concert with other regions such as the hypothalamic preoptic area that mediates autonomic components of thermoregulation [129]. Cold thermal information is relayed through the external lateral PBC to recruit shivering and nonshivering thermogenesis (e.g., metabolism of brown fat). Warm and hot temperature information is received in the dorsolateral PBC and conveyed by dynorphinergic neurons to inhibit thermogenesis. External lateral PBC neurons that express CGRP (calcitonin gene-related polypeptide) project to the amygdala and participate in malaise, cachexia, itch, chemosensory arousal, hyperalgesia and chronic pain. Inhibition of these nerves restored normal behaviours in animal models and generated the hypothesis that CGRP-expressing neurons in the PBC provide a multimodal threat signal that engages defensive behaviors [130]. A corollary is proposed that CGRP antagonists used for migraine [131] may be of therapeutic benefit to combat complaints that are enshrined as diagnostic criteria for ME/CFS and GWI.

## 14. Dysfunction in ME/CFS and GWI

The primary goal of the ascending arousal network is to instantaneously provide assessments of imminent threats for survival (Figure 4). Many midbrain and brainstem nuclei are activated. In addition, there is a general release of norepinephrine (LC), acetylcholine (PTN), and serotonin (MR) from ascending efferent axons that incite arousal and augment attention in the cerebral cortex. The PAG is a central hub for the assessment of predator-prey situations as well as stressful social encounters and fosters decisions about freeze, flight, fight, and faint responses. The MRF is activated during arousal and plays a modulatory role. Sensory inputs to IC contribute to sensory integration, orientation, and the startle response that is exaggerated in ME/CFS and GWI. The IC has high metabolic activity and may have impaired function when cerebral or local blood flow is reduced. IC is vulnerable to heavy metals and other toxic conditions. The PBC evaluates ascending sensory inputs for visceral interoception and nociception. The balance of glutamate vs. GABA plays a role in the development of systemic hyperalgesia and tenderness that are present in ME/CFS and GWI. Of particular relevance are malaise and sickness behaviors, and thermoregulatory control issues. The LC may be considered a motor nucleus for arousal as it activates many midbrain nuclei, and stimulates generalized sympathetic discharge for imminent activity.

**Figure 5:**
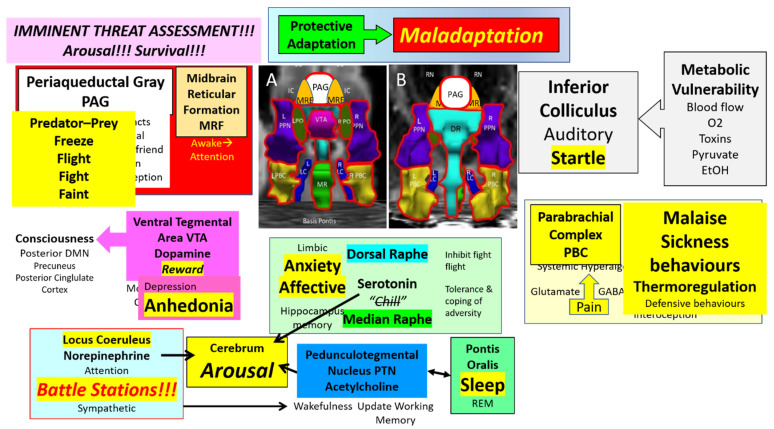
Midbrain ascending arousal network nuclei under stress. Prolonged or severe stressors can cause degeneration into maladaptation that tips the balance toward a dysfunctional state. Changes compared to Figure 4 emphasize potential alterations in ME/CFS and GWI. Stressors may have exaggerated consequences in particularly vulnerable populations. For example, VTA may balance between reward and motivation vs. anhedonia. DR and MR are poised between tolerance of aversive conditions and anxiety, affective and limbic system dysfunction. Glutamine and GABA balance the tolerance of pain vs. the development of systemic hyperalgesia. Arousal network nuclei with significant incremental changes in BOLD signal between days (∆) were outlined in red.

A theme in midbrain pathology is the imbalance between normal functions of homeostasis and wellbeing versus dysfunctional affect, mood, and sleep (Figure 5). Protective adaptation to chronic stressors may lead to maladaptation, for example with glutaminergic overload in the PBC leading to tenderness or galanin overexpression in LC that blunts arousal, cognition, and other active protective functions. The VTA is responsible for reward, motivation, and consciousness, but dysfunction is associated with anhedonia and depression. The DM and MR release serotonin to inhibit fight-flight responses, foster tolerance and coping to aversive stimuli and stimulate the hippocampus for long-term memory formation. Disruption affects the limbic system with anxiety and affective consequences. An imbalance between the cholinergic PTN that supports wakefulness versus the sleep-promoting activity of PO may lead to pathologic sleep problems. PTN monitors and refreshes the working memory that is required for the 2-back task used as a cognitive challenge in our paradigm. Chronic or severe stressor effects in each of these nuclei may reduce their function and release pathological responses of decreased arousal and effort, anhedonia, insomnia, increased interoception and pain, sympathetic overload, anxiety, and social withdrawal as part of sickness behaviors. The stressors may have a larger effect on the metabolically susceptible inferior colliculus than under usual circumstances which will augment startle and other aberrant responses. Underlying neuroimmune, autoimmune, metabolomic, or other mechanisms may all have direct effects that lead to dysfunction of these nuclei and the arousal system.

Sickness behaviors are adaptive responses to internal and external stressors in mammals. Headache phenotypes have been interpreted as an extension of these behaviors. Migraine, which is common in ME/CFS and GWI [132,133], may represent social withdrawal, motor quiescence, inhibition of sympathetic effects, lethargy, and apathy with excessive interoceptive signals [134]. In contrast, cluster headaches, which are uncommon in ME/CFS and GWI, may be interpreted as exteroceptive pain with the arousal of sympathetic fight-flight reactions, motor agitation, and restlessness. The molecular mechanisms of sickness behaviors are complex but may involve IL6, IL1-beta, INF-alpha, and other cytokines as well as modulation of arachidonic acid cytochrome P450 metabolites and phosphatidylinositol derivatives by eicosapentaenoic acid (EPA) and effects on cannabinoid CB2 receptors [135,136,137,138]. EPA may have some benefits for treating anxiety [139] and PTSD [140,141] but has small non-clinical effects compared to placebo in depression [142]. This provides the rationale for testing in ME/CFS [143] and GWI. Sickness behaviors may also involve disruptions of the anterior default mode network (DMN) that are correlated with systemic IL6 levels [144].

The dichotomy of exercise-induced changes in BOLD activation of the dorsal midbrain in ME/CFS vs. GWI provides new insights into PEM and indicates the two diseases have distinct pathological mechanisms that impact the same nuclei of the arousal network in but different ways. The upward and downward changes after exercise may not be as important as the distinction between ME/CFS and GWI. Both directions of change may be associated with the suite of dysfunctional effects in the dorsal midbrain, isthmus, arousal activation network and its connections that alter cognition, sleep, activity, affect, pain, and internal perception of “fatigue”.

## 15. Limitations

This approach has multiple limitations. Echoplanar imaging, especially at the base of the skull, is susceptible to structural distortion and artifacts that require careful correction [31]. The brainstem moves with each breath and heart beat which requires gating by simultaneous electrocardiograms. It is a challenge to portray the three-dimension orientation of the nuclei in the usual plane of MRI scanning. As a result, the cartoon images (Figure 1 and Figure 2) must be interpreted with extreme caution. Even though the Harvard atlas was defined by histological and white matter tracing, the range of human anatomical variability makes it difficult to ensure that the seed regions were in fact congruent with the underlying averaged data from our previous fMRI analysis. The variability in overlap is apparent by comparison to other atlases that are also based on histological sections and 3T MRI scans of the brainstem [38].

The small volume of the midbrain and smaller volumes of the nuclei make quantification difficult. 7T scanners may give better resolution. Combined tractography with region of interest studies is likely to delineate nuclei more precisely. Additional atlases are now available for seed regions such as the superior and inferior colliculi, red nucleus, amygdala, hypothalamic and thalamic nuclei that would be useful to extend the scope of these investigations. Measurement of global cerebral blood flow would also be useful to determine if flow was affected in the ME/CFS or GWI subjects. Other issues involve phenotyping the subjects. Multivariate methods would be beneficial in future studies to assess the influence of PTSD, anxiety, and systemic hyperalgesia as well as age, gender, ethnicity, and race. These studies do not identify any molecular mechanisms in the pathologies of ME/CFS and GWI, but rather suggest there are differences between the two diseases.

## 16. Conclusions

Review of the midbrain nuclei provides a novel perspective on potential neural pathologies affecting periaqueductal gray, parabrachial complex, inferior colliculus (startle), oculomotor and visual systems in threat assessment, anxiety, negative emotion, pain and tenderness, and other aspects of the ME/CFS and GWI clinical experiences. The data provide an initial framework to power future studies of postexertional malaise and midbrain dysfunction. This review is agnostic to whether midbrain lesions are primary for the pathogenesis of ME/CFS or GWI but is still consistent with metabolomic, autoimmune, and other etiologies that may have negative impacts on this highly vulnerable energy and oxygen-dependent organ. Mechanisms of cerebral or regional blood may place this region at particular risk. The extensive influence of the midbrain on life-sustaining neurobehaviours in psychological, psychiatric and neurological disorders is likely to revolutionize our understanding of brain diseases and open a fresh chapter for the investigation of ME/CFS and GWI. This is not a unifying hypothesis, though, because subtle differences in regional blood flow before and after exercise, and the opposite directions of change in ∆BOLD after exercise indicate that ME/CFS and GWI have different exercise-induced pathological mechanisms.

## Figures and Tables

**Table 1:** Diagnostic criteria. Changes in criteria of CFS, GWI, and fibromyalgia over time are indicated from left to right. The 1990 fibromyalgia definition was the only one to require an objective finding (tenderness to pressure or systemic hyperalgesia). Postexertional malaise is proposed to be the most discriminating symptom in CFS and SEID criteria. * includes chronic idiopathic fatigue and atypical depression.

FM 1990	Oxford 1991	CFS “Fukuda” 1994	Chronic Multi-Symptom Illness (CMI) 1998	GWI “Kansas” 2000	Canadian Consensus Criteria ME/CFS 2003, 2007	FM 2010, 2011, 2016	SEID 2015
		Disability/ Reduced Quality of Life			Disability/Reduced Quality of Life		Disability/ Reduced Quality of Life
Tenderness							
Widespread Pain		Myalgia Arthralgia	Musculoskeletal pain	Pain	Widespread Pain	Widespread Pain	
	Fatigue	Fatigue	Fatigue	Fatigue	Fatigue	Fatigue	Fatigue
		Sleep		Sleep	Waking unrefreshed	Waking unrefreshed	Waking unrefreshed
		Post-exertional malaise		Post-exertional malaise	Post-exertional malaise		Post-exertional malaise
		Cognition	Cognition	Cognition	Cognition	Cognition	Cognition
			Mood	Mood			
		Interoceptive symptoms					
		Headache Sore throat Sore lymph nodes		Neurological Gastrointestinal Respiratory Skin	Neurological Autonomic Neuroendocrine Immune	Somatic symptoms	Orthostatic Intolerance
Key features for diagnosis							
Pain + Tenderness	No exclusion *	Fatigue plus ≥4 of 8	≥1 chronic symptom in ≥2 categories	≥3 of 6 categories		Severity scores	Moderate or severe >50% of time
